# The Cardiovascular Conundrum in Ethnic and Sexual Minorities: A Potential Biomarker of Constant Coping With Discrimination

**DOI:** 10.3389/fnins.2021.619171

**Published:** 2021-05-19

**Authors:** Fausta Rosati, DeWayne P. Williams, Robert-Paul Juster, Julian F. Thayer, Cristina Ottaviani, Roberto Baiocco

**Affiliations:** ^1^Department of Developmental and Social Psychology, Faculty of Medicine and Psychology, Sapienza University of Rome, Rome, Italy; ^2^Department of Psychological Science, University of California, Irvine, Irvine, CA, United States; ^3^Department of Psychiatry and Addiction, University of Montreal, Montreal, QC, Canada; ^4^Department of Psychology, Faculty of Medicine and Psychology, Sapienza University of Rome, Rome, Italy; ^5^Functional Neuroimaging Laboratory, IRCCS Santa Lucia Foundation, Rome, Italy

**Keywords:** cardiovascular conundrum, minorities, discrimination, heart rate variability, hemodynamic profile

## Abstract

**Background:** A paradoxical profile of greater elevated sympathetic vasoconstriction (increased total peripheral resistance, TPR) and increased vagally-mediated heart rate variability (HRV) -the so-called Cardiovascular Conundrum- has been reported in African Americans (AAs) both at rest and in response to orthostasis. Whereas some authors have attributed this pattern to genetic factors, others have pointed to the potential role of coping with repeated racial discrimination.

**Objective:** To disentangle between these alternative explanations, we have examined the hemodynamic profile of another population that is likely to be exposed to episodes of discrimination, i.e., sexual minorities.

**Methods:** The first study was conducted on a sample of AAs and European Americans (EAs) with the aim of replicating previous results on the Cardiovascular Conundrum. In the second study, lesbian, gay, and bisexual (LGB) people, matched by age and sex with heterosexual participants, underwent a hemodynamic and autonomic assessment at rest and during an emotional (in the experimental group, both LGB-related and non LGB related), and a cognitive stressor.

**Results:** The first study confirmed a pattern of higher resting HRV, paired with higher TPR, in AAs compared to EAs. In the second study, compared to heterosexuals, the LGB group showed the Cardiovascular Conundrum pattern, characterized by greater HRV and higher TPR at baseline and a more vascular hemodynamic profile and prominent compensation deficit in response to both tasks, and particularly during the LGB-related emotional task. However, in LGB only, the vascular response was negatively correlated with perceived discrimination.

**Conclusion:** Present preliminary results are discussed in terms of maladaptive physiological consequences of exposure to chronic stress and the chronic use of dysfunctional emotion regulation strategies such as suppression.

## Introduction

Despite overwhelming evidence that African Americans have greater hypertension (Go et al., 2014) and related mortality and morbidity (Pool et al., 2017) than European Americans, the ethnic difference in hypertension still remains unexplained. Based on the reactivity hypothesis (Obrist, 1981), positing higher cardiovascular risk for physiological hyper-reactors, one would assume to find chronic sympathetic nervous system hyperactivation and parasympathetic withdrawal in African American. In the past decade, however, a series of studies have found a completely opposite picture, with African Americans being characterized by tonically higher heart rate variability (HRV), a measurement of parasympathetic autonomic function, compared to European Americans (reviewed in Hill and Thayer, 2019). This is surprising, given that low (not high) tonic HRV has been associated with a number of established and emerging modifiable and non-modifiable cardiovascular risk factors, including hypertension (e.g., Thayer et al., 2010). To make the picture even more complicated, this is also associated with higher total peripheral resistance (TPR; a measure of the amount of force affecting resistance to blood flow throughout the circulatory system) at rest (Brownlow et al., 2020 for a meta-analysis) and a more vascular (compared to myocardial) hemodynamic profile in response to stressors (e.g., Carnevali et al., 2019) in African American. The term “hemodynamic profile” describes the relationship between cardiac output (CO; a measure of the amount of blood the heart pumps in a minute) and TPR in the homeostatic regulation of blood pressure (Gregg et al., 2002). More vascular reactors respond to a stressor by increasing TPR more than CO, with the latter being predominantly increased by myocardial reactors, with the same ending point of increased blood pressure.

Considering that the baroreflex regulates both cardiac vagal tone and vascular resistance, such a pattern of both high HRV and higher TPR in African Americans represents a physiological enigma. Indeed, this has been referred to as the Cardiovascular Conundrum (Hill et al., 2015, 2017). Recent findings point to the role of constant effortful emotion regulation engaged by African Americans in response to daily discrimination (Hill et al., 2017; Thayer et al., 2020). A systematic review of the literature has suggested that perceived racial discrimination is linked with hypertensive status, being associated in particular with increased nighttime ambulatory blood pressure (BP), especially among African Americans (Dolezsar et al., 2014). In general, greater self-reported discrimination is coupled with lower resting HRV in African Americans (Hill et al., 2017), and this association appears to be moderated by rumination (Williams et al., 2017). For example, a previous study has shown that African Americans who expressed their anger had lower HRV and lower HRV recovery from a racially charged debate compared to their European Americans counterparts and compared to African Americans who inhibited their anger, which is considered a more socially appropriate response (Dorr et al., 2007). It has to be noted that while high tonic HRV is a measure of robust parasympathetic control on the heart and of the ability to engage in context-appropriate responses, phasic HRV suppression represents the withdrawal of cardiac vagal control and the activation of the defensive systems (Park et al., 2014). On the other hand, in a large, pooled dataset of 452 European Americans and 102 African Americans, greater use of reappraisal and suppression of anger were associated with greater HRV in African Americans but not in European Americans. Moreover, anger expression correlated with HRV in African Americans only (Thayer and Koenig, 2019). Notably, Thayer and Koenig (2019) found that cerebral blood flow in the anterior cingulate cortex was negatively associated with HRV in African Americans, whereas the opposite pattern emerged for European Americans. The authors conclude that the use of these habitual emotion regulation strategies may be associated with altered autonomic and central nervous systems coupling in African Americans (Thayer and Koenig, 2019). In a large sample of African American women (*N* = 208), only those reporting active coping with racism were characterized by a positive association between daily discrimination and hypertension, whereas the opposite pattern emerged for those characterized by low levels of active coping (Michaels et al., 2019). A number of recent studies provide support for the association between perceived discrimination or racism and poorer cardiovascular health in African Americans (e.g., metabolic syndrome in Cardel et al., 2020; several stress markers in Cedillo et al., 2020; urinary catecholamines in Homandberg and Fuller-Rowell, 2020).

Overall, the reviewed evidence suggests that the Cardiovascular Conundrum might emerge from the need to exert constant control over one’s anger (either with the use of rumination, suppression or reappraisal) in response to discrimination (Thayer et al., 2020). To investigate this hypothesis and rule out the contribution of the genetics of hypertension in African Americans (for a recent review see Zilbermint et al., 2019), one should investigate the same physiological pattern in a White population that is similarly exposed to discrimination. In this way, it would be possible to determine whether discrimination represents a key factor underlying ethnic disparities in cardiovascular functioning, regardless of the presence of “at risk” genetic polymorphisms putatively associated with specific ethnicities.

Sexual minorities appear to be the optimal population to study with this regard, as they are characterized by increased risk of stigma and prejudice (Herek, 2004). Through the *minority stress model*, Meyer (2003) conceptualized stigma as a source of psychosocial stress that is additive to the other stressors that are experienced by the majority of people and chronic because it depends on quite stable social and cultural structures. Minority stress processes range from distal objective stressors (e.g., discrimination, harassment, and victimization) to more proximal subjective stressors (e.g., expectations of rejections, vigilance, and internalized sexual stigma).

Based on the sexual minority stress model, several studies have shown the detrimental impact of sexual minority stress and stigma on lesbian, gay, and bisexual (LGB) people’s mental health (Kuyper and Fokkema, 2011; Lehavot and Simoni, 2011; Dürrbaum and Sattler, 2019; Baiocco et al., 2021). Only a few studies, however, examined the impact of sexual minority stress on outcomes such as cardiovascular function, and highlighted potential increased risk for health in sexual minorities when compared to the heterosexual population (Mays et al., 2018; Caceres et al., 2019). To date, none of these studies have assessed HRV. With a few exceptions, most studies assessed both minority stressors and health outcomes through interview or self-report methods, which made the results partially unclear. An exception is represented by the study of Cook and colleagues, who found that sexual orientation moderates the association between parental overprotection and stress biomarker profiles of acute and chronic stress responses, assessed by stress reactive cortisol and allostatic load (indexed using several neuroendocrine, immune, metabolic and cardiovascular biomarkers), respectively, suggesting underlying differential profiles of physiological stress processes among LGB and heterosexual individuals (Cook et al., 2018).

The present study had two interrelated objectives. The first was to replicate previous findings on the Cardiovascular Conundrum in African Americans, and we hypothesized to find a pattern of higher HRV associated with higher TPR at rest. The second was to examine if LGB people, who are similarly exposed to unfair treatment, show the same Cardiovascular Conundrum pattern as repeatedly found in African Americans. Specifically, we hypothesized (i) to find a more vascular HP in LGB compared to heterosexual people, particularly during reactivity to and recovery from the LGB-related task and that (ii) this pattern of more vascular profile as well as higher resting HRV would be positively associated with self-reported day-to-day minority stress (i.e., scores on the DHEQ).

## Materials and Methods

Hemodynamic profiles and HRV were assessed in two stigmatized social groups to better define the physiological concomitants of dealing with discrimination. Study 1 involved physiological profiling at rest in African American participants and focused on racial discrimination; Study 2 involved physiological profiles in response to social and cognitive stressors in LGB participants and focused on discrimination based on sexual stigma.

### Study 1

#### Participants

Participants were recruited via two methods: (1) an introductory level psychology course research pool for partial class credit; and (2) cash compensation for individuals’ participation outside of the research pool at The Ohio State University. Participants were recruited for the purposes of a larger study; however, results from these data have not been published elsewhere. Fifty-eight individuals (30 AAs and 28 EAs) were available for analyses. Participants were between the ages of 18–30, with an average age of 19.83 years old (*SD* = 2.2 years). Body Mass Index (BMI) for the full sample ranged from 19.25 to 47.27 Kg/m^2^ (*M* = 26.22, *SD* = 5.74). For African Americans, BMI ranged from 19.92 to 47.26 Kg/m^2^ (*M* = 27.47, *SD* = 6.68) and for European Americans, BMI ranged from 19.25 to 43.76 Kg/m^2^ (*M* = 24.96, *SD* = 4.39). Exclusion criteria, assessed via self-report questionnaires, were diagnosis of hypertension, heart disease, psychiatric disorder or habitual intake of drugs/medications affecting the cardiovascular system.

The Ohio State Institutional Review Board approved the study, and all participants signed written informed consent.

#### Procedure

We asked all participants not to smoke, undergo vigorous physical activity, or drink caffeine during the 6 h prior to the experiment. Participants then completed a 5 min baseline period, in which they sat in a resting position (spontaneous breathing) with the television displaying a blank, gray screen, and were instructed not to move or fall asleep.

#### Physiological Assessment

Beat-to-beat BP and HR were recorded from the non-dominant middle finger by using finger photoplethysmography (Finometer Pro, FMS, Finapres Measurement Systems, Amsterdam, The Netherlands; sampling rate: 200 Hz), tested against a mercury sphygmomanometer. During physiological assessment, participants were in a seated position with their non-dominant hand lying on a table. Raw pulse wave files were converted to text files by Beatscope Easy. Text files were then processed using a custom-made LabVIEW software (LabVIEW8.5, National Instruments, Austin, Texas, United States) to calculate mean indices of systolic BP (SBP), root mean square of successive difference (RMSSD), as well as TPR, and stroke volume (SV) derived using the Modelflow method. RMSSD reflects vagal regulation of HR (Task Force of the European Society of Cardiology and the North American Society of Pacing and Electrophysiology, 1996) and is less susceptible to respiratory influences compared to frequency-domain measures of HRV (Penttilä et al., 2001).

#### Data Analysis

All statistical tests were conducted using SPSS (ver. 20, IBM Chicago, IL, United States). Independent samples *t*-tests were conducted to analyze differences in baseline physiological variables between AAs and EAs. Group differences in age, BMI, and sex were also examined. RMSSD was natural log-transformed to fit assumptions of linear analyses. Given extensive literature and meta-analyses (e.g., Hill et al., 2015; Brownlow et al., 2020) on baseline physiological differences between AAs and EAs, all tests were one-tailed and significance levels were evaluated using an alpha of 0.05. Effect sizes are reported as Cohen’s *d.*

## Results

AAs and EAs did not significantly differ on BMI [*t*(56) = 1.69, *p* = 0.099], sex (χ^2^ = 2.01, *p* = 0.184), or age [*t*(56) = 1.31, *p* = 0.196].

See Table 1A for all resting cardiovascular and hemodynamic parameters in AAs and EAs. AAs showed significantly greater SBP [*t*(56) = −1.87, *d* = 0.50, *p* = 0.035], RMSSD [*t*(56) = −1.87, *d* = 0.49, *p* = 0.033], and TPR [*t*(56) = −2.36, *d* = 0.65 *p* = 0.011] compared to EAs. It is important to note that these results remain significant if ANOVAs controlling for BMI as a covariate are performed instead of *t-*tests.

**TABLE 1:** Mean and standard deviations of resting cardiovascular and hemodynamic parameters in African Americans (AAs) compared to European Americans (EAs) (study 1) and in Lesbian, Gay and Bisexuals (LGB) compared to heterosexual (HS) participants (study 2).

**Table 1A—study 1**	**AAs (*n* = 30)**	**EAs (*n* = 28)**	***t***	***p***
MAP (mmHg)	70 ± 10	64 ± 5	−2.51	0.015^*^
SBP (mmHg)	122 ± 12	116 ± 10	−1.87	0.035^*^
IBI (ms)	840.99 ± 91.33	828.46 ± 141.11	−0.40	0.344
HR (bpm)	72.13 ± 7.67	74.49 ± 12.82	0.853	0.397
RMSSD (ms | natural log)	3.98 ± 0.40	3.76 ± 0.50	−1.87	0.033^*^
SV (ml)	91.62 ± 25.32	95.43 ± 20.58	0.63	0.267
CO (l/min)	6.50 ± 1.52	7.00 ± 1.51	1.26	0.213
TPR (PRU)	806.69 ± 265.31	673.33 ± 143	−2.36	0.011^*^

**Table 2A—study 2**	**LGB (*n* = 19)**	**HS (*n* = 20)**	***t***	***p***

MAP (mmHg)	106 ± 28	89 ± 10	2.45	0.019^*^
SBP (mmHg)	142 ± 33	123 ± 12	2.39	0.022^*^
HR (bpm)	76.99 ± 8.4	81.58 ± 14.2	1.22	0.231
RMSSD (ms)	41.44 ± 10.8	33.2 ± 13.9	2.05	0.047^*^
CO (l/min)	5.15 ± 1.5	5.42 ± 1.2	0.61	0.549
TPR (PRU)	1519.63 ± 778.5	600.91 ± 634.4	4.05	<0.001^*^

SBP, systolic blood pressure; IBI, inter-beat-intervals; RMSSD, root mean square of successive differences; SV, stroke volume; TPR, Total Peripheral Resistance; PRU, Peripheral Resistance Units; MAP, Mean Arterial Pressure; HR, Heart Rate; CO, Cardiac Output. Significant p-values bolded. ^*^p < 0.05.

### Study 2

#### Participants

Recruitment occurred through the snowball sample technique, as well as using a flyer posted on social media and the “Be as you are” research and clinical center (Baiocco and Pistella, 2019). Inclusion criteria for the study were: (1) self-identifying as LGB; (2) not having diagnosis of hypertension, heart disease, and psychiatric diagnosis; and (3) not using any drugs/medications that might affect cardiovascular function. Of the 40 people who took part, 1 was excluded due to Portapres device malfunction. A total of 19 LGB (mean age: 35.42 (10.88) years; BMI: 25.33 (5.45) Kg/m^2^) and 20 heterosexual individuals (mean age of 35.26 (10.14) years, BMI: 25.78 (3.42) Kg/m^2^) participated in the study. The LGB group was composed by 8 cisgender women who self-identified as lesbians (*n* = 6) or bisexuals (*n* = 2) and 11 cisgender men who self-identified as gay (*n* = 10) or bisexual (*n* = 1). To discount the effects of genetics (and potentially of diet/lifestyle) on the conundrum in African Americans, all participants were Caucasians. Most participants were of middle socio-economic status, 2 participants of low- and 2 of high-socio-economic status. The educational level varied from high school diploma (*n* = 15) to bachelor’s (*n* = 9) or higher degree (*n* = 15).

#### Procedure

To ensure data accuracy, participants were asked not to drink coffee and alcohol, not to smoke cigarettes and to avoid strenuous exercise for 2 h prior to the appointment. The experiment took place at the premises of the “Be as you are” clinical service of the Department of Developmental and Social Psychology, Sapienza University of Rome, in a quiet room with a closed door. After informed consent procedures, the continuous BP cuff was attached on the index finger of participants’ dominant hand, while participants were in a seated position with their hands lying on a table. The experimental protocol started with a baseline period of 2 min followed by three experimental conditions: (1) a cognitive task, consisting in performing simple arithmetic operations and verbally report the results; (2) an emotional stress induction task, consisting in asking participants to verbally report a stressful episode of their life; and -for the LGB group only- (3) a sexual minority emotional stress induction task, consisting in asking participants to verbally report a stressful episode related to their LGB identity. The order of the stressful tasks was randomized for each participant. The cognitive task lasted 2 min, while each of the emotional tasks lasted 5 min. Each task was followed by a recovery period of 5 min, during which participants were invited to remain silent and just leaf through a content-neutral magazine (Jennings et al., 1992).

#### Experimental Task Instructions

The instructions for the cognitive task were: “*Now I will ask you to count backwards out loud by subtracting 5 units from the number that I will tell you. As an example: The starting number is 285. The following numbers are: 280, 275, 270, 265, and so on. Is everything clear? I will tell you when to stop. Now it is your turn. The starting number is 253*.”

The instructions for the emotional stress induction task were: “*Now I ask you to tell me about a stressful episode in your life* (for the LGB participants we specified that the stressful episodes should not be related to their sexual minority identity). *Let’s take an example. You could tell about a job interview that went wrong or a theft you suffered, a difficult time from an economic point of view. Take your time to think about it. Tell me when you are ready*.”

The instructions for the sexual minority emotional stress induction task were: “*Now I ask you to tell me about a stressful episode in your life which is related to your LGB identity. Let’s take an example. You could talk about an episode of discrimination at work, mockery or exclusion by your peers because you are LGB. Take your time to think about it. Tell me when you are ready*.”

#### Questionnaires

##### Sociodemographic Variables

The survey included several sociodemographic questions, to obtain information such as age, gender identity, sexual orientation, ethnicity, socio-economic status, and educational level. Exclusion criteria, assessed via self-report questionnaires, were diagnosis of hypertension, heart disease, psychiatric disorder or habitual intake of drugs/medications affecting the cardiovascular system.

##### Minority Stress Experiences

The *Daily Heterosexist Experiences Questionnaire* (DHEQ; Balsam et al., 2013) was used to measure day-to-day minority stress experienced by participants. For the purpose of this study, we used 5 of the 10 subscales of the questionnaire, assessing negative experiences related to gender expression (e.g., Being harassed in bathrooms because of your gender expression), vigilance (e.g., Watching what you say and do around heterosexual people), discrimination (e.g., Being treated unfairly in stores or restaurants because you are LGB), victimization (e.g., Being punched, hit, kicked, or beaten because you are LGB), and isolation (e.g., Difficulty finding a partner because you are LGB). Participants were invited to answer the following question: “How much has this problem distressed or bothered you during the past 12 months?” by using a 6-point Likert scale ranging from 0 (did not happen/not applicable to me) to 5 (it happened, and it bothered me extremely). Previous studies conducted in the Italian context showed good internal reliability of the scales (*blinded for peer review*).

##### Depressive Rumination

The Ruminative Response Scale (RRS; Nolen-Hoeksema and Morrow, 1991) was administered to exclude group differences in trait rumination, given the well-established effects of this trait on HRV and hemodynamic parameters (Ottaviani et al., 2016, 2017). The RRS measures how often people engage in responses to depressed mood that are self-focused (e.g., I think “Why do I react this way?”), symptom-focused (e.g., I think about how hard it is to concentrate), and focused on the possible consequences and causes of one’s mood (e.g., I think “I won’t be able to do my job if I don’t snap out of this”).

#### Physiological Assessment

Noninvasive continuous measurement of beat-to-beat BP was obtained throughout the study with the Portapres II (FMS; The Netherlands; sampling rate: 200 Hz) device, tested against a mercury sphygmomanometer. The BeatScope^®^ software, which is based on the Modelflow^®^ algorithm and corrects for age, height, and weight, was used to derive heart rate (HR), inter-beat intervals (IBIs), mean arterial pressure (MAP), cardiac output (CO), and TPR. Following the orthogonal, physiologically grounded model proposed by Gregg and colleagues for both reactivity and recovery periods, participants are described using two independent parameters: the way in which they respond, i.e., “more vascular or more myocardial” (hemodynamic profile; HP) and “the extent to which an increase of TPR compensates for an increase in CO and vice versa” (compensation deficit; CO) (Gregg et al., 2002). The model: (i) takes into account the multiplicative relationship between CO and TPR (Guyton, 1987); (ii) is based on the assumption that HP and CD are orthogonal; and (iii) uses ratio scores instead of difference scores of reactivity (see James et al., 2012 for a meta-analysis). The equation used to address the concept of hemodynamic profile was: log(CO)r + *log(TPR)_*r*_ = log(MAP)_*r*_*, where “*r*” in the equation refers to the ratio of task to baseline values for reactivity and to the ratio of resting to baseline for recovery.

Then, a 45° rotation of the two-dimensional space formed by the cardiac output and total peripheral resistance reactivity dimensions was performed to achieve the orthogonal relationship between HP and CD (see Gregg et al., 2002 for further methodological details). The outcome of the model is a continuous variable in which greater HP values correspond to more vascular responses and greater CD values indicate that increased TPR is not compensated by a proportionate decrease in CO (see Gregg et al., 2002 for methodological details).

HRV was assessed by computing the RMSSD: IBIs were transferred to Kubios HRV software for RMSSD analysis and artifact detection (Tarvainen et al., 2014). Ectopic beats were corrected using the “automatic correction” function, in which artifacts are detected from a time series consisting of differences between successive RR intervals.

#### Data Analysis

All data are expressed as means (SD). Differences at *p* < 0.05 were regarded as significant. Data processing was performed with the software modules of SPSS 25 (IBM). MAP, HR, RMSSD, CO, and TPR, HP, CD, and scores on RRS were treated as continuous variables.

Assumptions for normality were tested for all continuous variables using the Shapiro-Wilk test. Differences between the two groups (LGB vs. heterosexuals) in age, sex distribution, BMI, years of education, economic status, and levels of dispositional depressive rumination were analyzed by *t*-tests and χ^2^-tests. The variables that differed significantly between groups were included as covariates in all the subsequent analyses.

To test for group differences in reactivity and recovery for the shared two experimental conditions, a series of 2 (task: emotional vs. cognitive) ×2 (group: LGB vs. heterosexuals) general linear models (GLMs) were performed on HP and CD during reactivity and recovery. For the LGB group only, further analyses were conducted to test for differences in hemodynamics between the LGB-related and LGB-unrelated emotional task. To do so, a 2 (task: LGB-related vs. LGB-unrelated) ×2 (time: reactivity vs. recovery) GLMs were executed on HP and CD.

Lastly, Spearman’s correlations between scores on the DHEQ, resting RMSSD, and HP and CD during reactivity to and recovery from the LGB-related task (LGB group only) were performed.

#### Results

Each dependent variable was normally distributed. No group differences due to potential confounders (i.e., age, sex distribution, BMI, years of education, economic status, and levels of dispositional depressive rumination) emerged (all *p*s > 0.05). In the LGB group, scores on the DHEQ ranged from 1.07 to 2.33 (*M* = 1.55, *SD* = 0.35), and scores on the RRS ranged from 1.09 to 2.68 (*M* = 1.85, *SD* = 0.45).

Table 2A depicts resting cardiovascular and hemodynamic parameters in LGB and heterosexual participants. The two groups were significantly different in terms of MAP, TPR, and RMSSD at baseline with LGB having a higher MAP (*t* = 2.45, *p* = 0.019; *d* = 0.78), TPR (*t* = 4.05, *p* < 0.0001; *d* = 1.29) and RMSSD (*t* = 2.05, *p* = 0.047; *d* = 0.66) compared to heterosexuals. No differences emerged for HR and CO. This replicated the Cardiovascular Conundrum pattern of higher RMSSD and higher TPR found in AAs in Study One.

**FIGURE 1:**
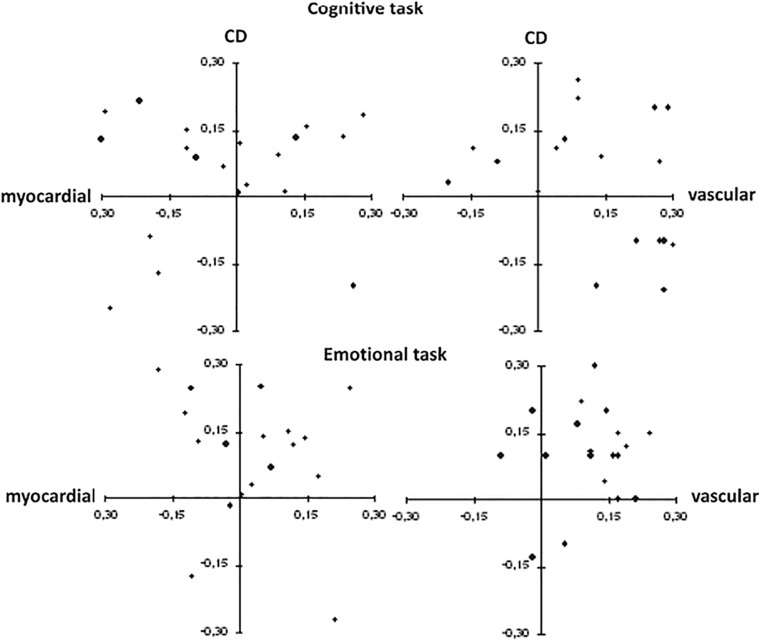
Scatterplots for hemodynamic profile and compensation deficit during each task in heterosexual (right panel) and lesbian, gay and bisexual (left panel) participants. Each point represents each participant’s hemodynamic profile (HP) and compensation deficit (CD). A “more vascular” profile is associated with more positive values along the *x*-axis and a “more myocardial” profile is associated with more negative values along the *x*-axis. A “higher deficit” in compensating is associated with more positive values on the *y*-axis and a “lower deficit” in compensating is associated with more negative values on the *y*-axis.

Figure 1 shows the relationship between hemodynamic profile and compensation deficit scores in the two groups for the different tasks. As for HP reactivity, main effects of Task, *F*(1, 37) = 6.26, *p* = 0.017; η *p*^2^ = 0.15, and Group, *F*(1, 37) = 5.69, *p* = 0.022; η*_*p*_*^2^ = 0.13 emerged, while the Task X Group interaction was not statistically significant. Pre-planned comparisons showed that, irrespective of task, LGB had a more vascular HP compared to heterosexual participants and that the emotional task was characterized by a more vascular pattern of response compared to the cognitive task.

A similar pattern of results emerged when CD was considered. The GLM yielded main effects of Task, *F*(1, 37) = 20.39, *p* < 0.0001; η*_*p*_*^2^ = 0.36, and Group, *F*(1, 37) = 4.30, *p* = 0.045; η*_*p*_*^2^ = 0.10 but no significant Task X Group interaction. As shown by pre-planned comparisons, LGB had a “higher deficit” in compensating compared to heterosexual participants and the emotional task was characterized by a higher CD compared to the cognitive task.

The GLMs having HP and CD during recovery from the tasks as outcomes did not yield any significant main effect or interaction.

**FIGURE 2:**
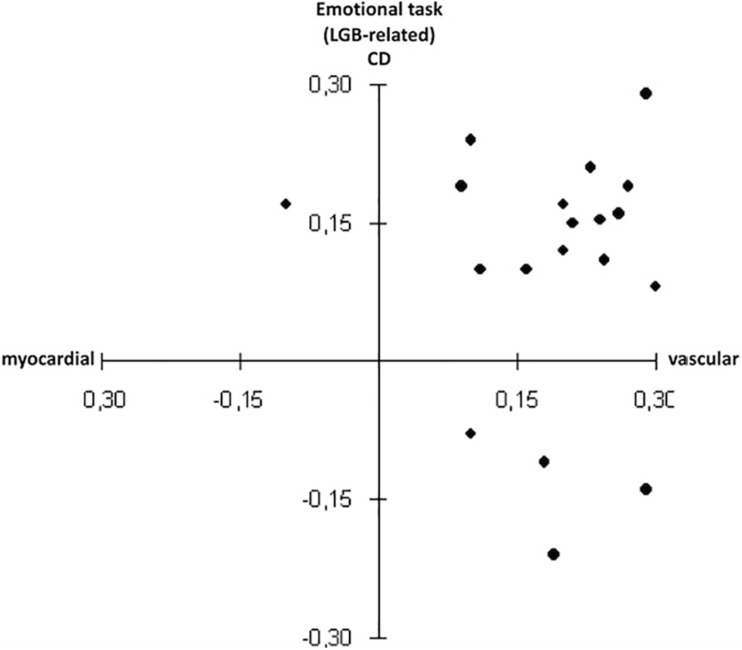
Scatterplots for hemodynamic profile and compensation deficit during the lesbian, gay and bisexual (LGB)-related task in the LGB group.

When the difference between LGB-related vs. LGB-unrelated emotional task in HP and CD for the LGB group was considered, the GLM yielded a marginally significant Time x Task interaction for HP, *F*(1, 18) = 4.04, *p* = 0.06; η*_*p*_*^2^ = 0.18. Pre-planned comparisons showed that a more vascular hemodynamic response emerged for reactivity to the LGB-related (see Figure 2) compared to the LGB-unrelated task (*t* = 5.30; *p* < 0.0001), whereas no differences emerged for the recovery phase.

Spearman’s correlations yielded significant inverse associations between scores on the day-to-day minority stress (DHEQ) and reactivity to the LGB-related task for both HP (*rho* = −0.59; *p* = 0.008) and CD (*rho* = −0.48; *p* = 0.037). Higher RMSSD at rest was also marginally associated with “higher deficits” in compensating during the LGB-related task (*rho* = 0.41; *p* = 0.07). No other significant correlations emerged.

## Discussion

The aim of this study was to preliminarily investigate whether the dysfunctional physiological profile found in African Americans is seen also in other minority groups subjected to discrimination, such as LGB. First, study 1 replicated previous reports on the presence of a so-called Cardiovascular Conundrum in African Americans, that is a pattern of high resting HRV coupled with high TPR, that is likely due to a dysfunction at the baroreflex level (Hill et al., 2015 for a meta-analysis of ethnic differences in HRV and Brownlow et al., 2020 for a meta-analysis on ethnic differences in TPR). Study 1 did not include a stressor because the Conundrum was originally found at rest, however, the fact that resting high HRV is also associated with a more prominent vascular hemodynamic reactivity to physical (i.e., orthostasis) and emotional (i.e., anger recall) stressors in African Americans has been previously reported (Carnevali et al., 2019).

As hypothesized by Thayer and Koenig (2019), we looked at whether this Cardiovascular Conundrum could be due to chronic exposure to a psychosocial stressor such as discrimination. Remarkably, when we looked at sexual minorities instead of ethnic minorities, the pattern was the same both at baseline and during confrontation with an emotional task. First, present data preliminarily suggest that when compared to heterosexual individuals, LGB people are characterized by higher HRV and higher TPR at rest after controlling for a number of potential confounders. Despite showing greater HRV at baseline, the LGB group showed a prominent vascular hemodynamic profile and compensation deficit during emotional tasks such as the recall of personal (both LGB- and non LGB-related) episodes, but not during a mathematical task. These results replicate and extend our prior findings of the Cardiovascular Conundrum pattern in African Americans compared to European Americans (Hill et al., 2015; Carnevali et al., 2019; Brownlow et al., 2020). Furthermore, they suggest that genetic factors are unlikely to account for this unique cardiovascular pattern.

To date, findings on cardiovascular health disparities in LGB are inconsistent. And yet, a recent review concluded that sexual minorities are at increased risk for cardiovascular disease (Caceres et al., 2017). As previously described with regards to ethnic minorities, this has been ascribed to hyperreactivity to stressors (see for example Juster et al., 2019 for differences between men and women). The present study took a step further, looking at the underlying hemodynamic profiles of cardiovascular reactivity and found that -compared to that of heterosexuals- the pattern shown by LGB participants was more vascular in nature. This is crucial information, given that elevated BP driven by TPR, compared to CO, has been linked to increased risk of cardiac events and mortality (Julius, 1988).

The associations between discrimination, reports or lack thereof of such discrimination and psychophysiological responses is complex. Whereas some studies have reported that discrimination is associated with deleterious physiological responses such as greater BP (Pascoe and Richman, 2009; Dolezsar et al., 2014), others have found that reports of discrimination associated with salubrious physiological responses such as greater HRV (Kemp et al., 2016). In addition, some studies have reported that fewer reports of discrimination are associated with greater physiological responses such as greater BP (Krieger and Sidney, 1996).

Notably, in the present study, a more vascular pattern during the recall of LGB-related episodes emerged in those who reported fewer daily experiences of discrimination, victimization, isolation, and so on (i.e., scores on the DHEQ). While counterintuitive, this result is not uncommon to find in the literature. For example, Christian and colleagues also found an inverse association between incidences of discrimination and TPR in pregnant African Americans, while this was not true for European Americans (Christian et al., 2020). In a larger study, working-class African American adults (*N* = 1,974) who typically accepted unfair treatment and had reported no experience of racial discrimination had higher systolic BP compared to those who challenged unfair treatment and reported experiencing racial discrimination (Krieger and Sidney, 1996). Similarly, in studying another minority ethnic group in the United States, Rodriguez and colleagues have found that a dysfunctional pattern of nocturnal non-dipping BP was particularly present in black-Hispanic participants with low perceived racism compared to those with high perceived racism (Rodriguez et al., 2016). The authors interpreted this result in terms of internalization of racism, where those with higher perceived racism may be “more proactive against discrimination and possibly less likely to internalize the associated stress” (Rodriguez et al., 2016). Interestingly, Frost et al. (2015) found that only externally rated minority stressors such as prejudice events -but not self-appraised self-exposure- predicted physical health problems, such as hypertension in LGB people. Such current and reviewed findings highlight the complex relationships among discrimination, reports of discrimination, and psychophysiological responses such that deleterious effects and salubrious effects may co-occur in the same individuals. Future studies are needed to further explicate these associations as effects may be non-linear or compensatory in nature.

*John Henryism* is considered a high-effort strategy to cope with discrimination (James et al., 1983). Current results suggest that such strategy is likely to involve processes that take place outside the individual’s awareness. It is mandatory to conduct further studies examining whether the Cardiovascular Conundrum is associated with a specific coping strategy that is peculiar of individuals who are subject to discrimination without being fully aware of it.

There are some limitations that need to be mentioned. In the second study, the number of participants was limited and *post-hoc* power analysis pointed to some of the analyses being underpowered (1 − β > 0.60); thus, replication with a larger sample is warranted before we can answer the research question “Is the Cardiovascular Conundrum a marker of constant coping with discrimination?” Moreover, the present study focused on the experience of being potentially subjected to discrimination without specifically assessing subjective levels of stress or coping strategies in response to it such as reappraisal and anger suppression. Based on prior work, however, we can speculate that suppression would be associated with higher HRV in minority groups only, therefore playing an important role in the evolution of the Cardiovascular Conundrum (Thayer and Koenig, 2019). Also, the cognitive and emotional tasks used in Study 2 had different durations (2 vs. 5 min) which may have biased the results. Lastly, it has to be noted that although estimation of HRV derived from photoplethysmographic technique is not considered the golden standard, studies have shown that HRV measures can be accurately derived using such technique in healthy subjects under ideal conditions (e.g., Lu et al., 2009).

Limitations notwithstanding, the current results may inform the interpretation of previous research on the Cardiovascular Conundrum in African Americans, pointing to the presence of this physiological pattern in other populations and suggesting a plausible underlying mechanism related to emotion regulation as opposed to genetic factors. Large scale data are needed to draw causal inferences on this complex and intriguing phenomenon.

## Data Availability Statement

The raw data supporting the conclusions of this article will be made available by the authors, without undue reservation.

## Ethics Statement

The studies were reviewed and approved by the Ohio State Institutional Review Board at The Ohio State University (Study 1) and Research Ethics Committee of the Department of Developmental and Social Psychology, Sapienza University of Rome, Rome, Italy (Study 2). The patients/participants provided their written informed consent to participate in this study.

## Author Contributions

JT, CO, DW, and RB contributed to the conceptualization of the study. FR and DW conducted the study and analyzed the data. CO wrote the initial draft of the manuscript. DW also contributed to writing elements of the manuscript. All authors contributed to the interpretation of the results, provided critical feedback, helped shape the analysis and manuscript, and approved the submitted manuscript.

## Conflict of Interest

The authors declare that the research was conducted in the absence of any commercial or financial relationships that could be construed as a potential conflict of interest. The reviewer SB declared a past publication with one of the author JT to the handling editor.
